# Microbiological Spectrum and Antimicrobial Resistance Patterns of Cerebrospinal Fluid Isolates from Neurosurgical Intensive Care and Ward Patients: A 10-Year Single-Center Study

**DOI:** 10.3390/jcm15187236

**Published:** 2026-09-17

**Authors:** Boran Urfalı, Burçin Özer

**Affiliations:** 1 Department of Neurosurgery, Tayfur Ata Sökmen Faculty of Medicine, Hatay Mustafa Kemal University, 31060 Antakya, Hatay, Türkiye; 2 Department of Medical Microbiology, Tayfur Ata Sökmen Faculty of Medicine, Hatay Mustafa Kemal University, 31060 Antakya, Hatay, Türkiye; burcinozer@mku.edu.tr

**Keywords:** neurosurgical patients, CSF, antimicrobial resistance, meningitis, ventriculitis, intensive care unit

## Abstract

**Background/Objectives**: Central nervous system (CNS) infections in neurosurgical patients remain a serious cause of morbidity and mortality, particularly in healthcare-associated settings. The microbiological profile and antimicrobial resistance patterns of these infections vary between institutions and regions. This study aimed to evaluate the distribution of cerebrospinal fluid (CSF) pathogens and their antimicrobial susceptibility profiles in neurosurgical patients treated at a tertiary care hospital in Hatay, Türkiye. **Methods**: This retrospective cross-sectional study included 447 patients with CSF culture and antimicrobial susceptibility results obtained between January 2015 and June 2025. **Results**: Overall, 17.2% of patients had positive CSF cultures, with significantly higher positivity in intensive care unit (ICU) patients compared with ward patients (30.2% vs. 12.7%, *p* < 0.001). The most frequently isolated pathogens were *Acinetobacter baumannii*, coagulase-negative staphylococci, and *Klebsiella pneumoniae*. Gram-negative organisms were more common in ICU patients (70.2%), whereas Gram-positive organisms predominated in ward patients (62.5%) (*p* = 0.001). No significant association was found between primary neurosurgical diagnosis and specific bacterial species (*p* > 0.05). However, Gram-negative predominance was observed in intracranial mass cases, while hydrocephalus was mainly associated with Gram-positive organisms. High antimicrobial resistance rates were observed among Gram-negative isolates, including carbapenem resistance (40.9%) and amikacin resistance (45.5%). Glycopeptides displayed activity against all Gram-positive isolates. **Conclusions**: The findings of this study support the importance of updated antimicrobial stewardship and further prospective multicenter studies to improve infection management in neurocritical care settings.

## 1. Introduction

Central nervous system (CNS) infections, including meningitis and ventriculitis, remain important causes of morbidity and mortality worldwide. Despite major advances in antimicrobial therapy, healthcare-associated CNS infections still represent a major challenge in clinical practice. Delayed diagnosis and inappropriate treatment may lead to severe neurological complications, prolonged hospitalization, and increased mortality. Microbiological examination of cerebrospinal fluid (CSF) is essential for the diagnosis of bacterial CNS infections, and CSF culture is still considered the gold standard for pathogen identification [1]. Early diagnosis together with appropriate empirical antimicrobial therapy plays a critical role in patient management. Treatment protocols are generally adjusted according to culture results and antimicrobial susceptibility testing.

With the development of neurosurgical procedures, healthcare-associated CNS infections have become an important clinical problem. Previous studies have reported incidence rates ranging from 0.3% to 7.4%, while mortality rates may reach 8–34% despite treatment [2,3]. Procedures such as craniotomy, CSF shunting, and external ventricular drain (EVD) placement are recognized risk factors for post-neurosurgical meningitis and ventriculitis [4,5]. Reported rates of post-neurosurgical meningitis vary between 0.3% and 10% [6]. Although these infections represent only a small proportion of all nosocomial infections, they are associated with high morbidity, mortality, and serious therapeutic challenges.

The microbiological profile of neurosurgical CNS infections differs from that of community-acquired meningitis. While community-acquired infections are mainly caused by pathogens such as *Streptococcus pneumoniae* and *Neisseria meningitidis*, hospital-associated infections are increasingly linked to opportunistic and multidrug-resistant (MDR) microorganisms [7]. Coagulase-negative staphylococci (CoNS), *Acinetobacter baumannii*, *Pseudomonas aeruginosa*, and *Klebsiella pneumoniae* are also among the most frequently reported pathogens in neurosurgical settings [7,8,9,10,11].

Antimicrobial resistance (AMR) has become a major global health threat, particularly in hospital-associated infections. Recent surveillance studies have shown increasing carbapenem resistance rates among CSF isolates, especially in *A. baumannii* and *K. pneumoniae*. In some reports, meropenem resistance rates reached 85.6% in *A. baumannii* and 64.1% in *K. pneumoniae* isolates obtained from CSF samples in China [12]. Treatment of CNS infections caused by MDR bacteria is difficult because many antimicrobial agents have limited penetration through the blood–brain and blood–CSF barriers. Therefore, combination antimicrobial therapies are often required in severe infections [13]. In Türkiye, increasing resistance rates among Gram-negative bacteria, particularly carbapenem-resistant *K. pneumoniae* and MDR *A. baumannii*, have also been reported [14,15]. These resistant pathogens are associated with prolonged hospitalization, limited treatment options, increased healthcare costs, and poor clinical outcomes. Although several studies describing healthcare-associated CNS infections have been published from different regions of Türkiye, most were limited to relatively short study periods or focused on specific patient groups [16,17,18,19]. Therefore, updated data from different tertiary referral centers are needed to determine whether bacterial epidemiology and antimicrobial resistance patterns are consistent across the country and to improve the evidence base for empirical treatment recommendations.

Therefore, the present study aimed to evaluate the microbiological distribution and antimicrobial resistance profiles of CSF isolates obtained from neurosurgical intensive care unit (ICU) and ward patients at a tertiary university hospital in Hatay, Türkiye, between 2015 and 2025.

## 2. Materials and Methods

This retrospective cross-sectional descriptive study was conducted at the Faculty of Medicine Hospital, Hatay Mustafa Kemal University, Türkiye. To minimize selection bias, no sampling was performed; all 447 neurosurgical ICU and ward patients from whom CSF samples were obtained and tested between January 2015 and June 2025 were included. The hospital is an approximately 600-bed tertiary care center providing healthcare services to a population of approximately 1.6 million in Hatay Province, as well as to patients referred from conflict-affected regions in Syria. Only antimicrobial susceptibility testing (AST) data for isolates recovered from these cultures were analyzed.

CSF samples were collected under sterile conditions from various clinical departments and processed immediately in the microbiology laboratory. Standard bacterial culture was performed using 5% sheep blood agar and chocolate agar media, while fungal cultures were performed using Sabouraud dextrose agar. Identification was performed using the VITEK 2 automated microbiology system (bioMérieux, Marcy-l’Étoile, France). Antimicrobial susceptibility testing (AST) was performed using the VITEK 2 system and interpreted according to the European Committee on AST (EUCAST) breakpoints [20]. When required, susceptibility results were confirmed using the broth microdilution method, particularly for antibiotics such as carbapenems and colistin. For quality control, isolates of *E. coli* ATCC 25922, *P. aeruginosa* ATCC 27853, and *S. aureus* ATCC 25923 were used. Isolates were categorized as susceptible, intermediate (increased exposure), or resistant according to the EUCAST criteria. Persistent isolation was defined as repeated recovery of the same bacterial species from CSF cultures obtained from the same patient during subsequent sampling episodes.

Descriptive statistics were used for data analysis. Statistical analyses were performed using IBM SPSS Statistics for Windows, version 29.0 (IBM Corp., Ar-monk, NY, USA) and MedCalc Statistical Software, version 23.6.1 (MedCalc Software Ltd, Ostend, Belgium). Continuous variables are presented as the mean ± standard deviation, while categorical variables are presented as the number (*n*) and percentage (%). Age at admission of the ward and intensive care unit groups was compared using Student’s *t*-test. Categorical variables of the ward and intensive care unit groups were compared using Pearson’s chi-square test or Pearson’s chi-square exact test, as appropriate. The overall distribution of diagnoses was evaluated using chi-square/Fisher’s exact tests. Since the overall diagnosis comparison was statistically significant, group proportions for individual diagnosis categories were additionally compared using the two-proportion z-test. The 95% confidence intervals for the proportions were calculated using the Wilson score method. In the histopathological classification analysis, only patients with evaluable histopathological results were included, and percentages were calculated based on these patients. Antimicrobial susceptibility testing results were deduplicated at the patient–organism–antibiotic level. Only antimicrobial agents with a cumulative test count of *n* ≥ 4 among the tested isolates were presented. Antimicrobial susceptibility percentages were calculated using the number of tested isolates for each agent as the denominator. A *p*-value of <0.05 was considered statistically significant.

This study was approved by the Non-Interventional Clinical Research Ethics Committee of Hatay Mustafa Kemal University on 9 July 2025 (approval number: 11). As this was a retrospective study based on routinely collected laboratory data, informed consent was not required. During the preparation of this manuscript, the authors used ChatGPT-5.6 Sol for the purposes of English language editing. The authors have reviewed and edited the output and take full responsibility for the content of this publication.

## 3. Results

The baseline demographic characteristics of the analyzed patients by hospital unit are summarized in Table 1. A total of 447 patients were included in this study. Of these, 116 (26%) were treated in the ICU and 331 (74%) were managed in the ward service. Of these, 195 (43.6%) were female and 252 (56.4%) were male. The difference in gender distribution was not statistically significant (*p* = 0.23). The mean age of the cohort was 36.1 ± 14.8, and ranged from 0 to 89 years. There was no statistically significant difference in age between patients hospitalized in the ward and those admitted to the ICU (mean age: 35.5 ± 14.6 vs. 37.7 ± 15.2 years, respectively; *p* = 0.41).

The distribution of underlying diagnoses differed significantly between ward and ICU patients (*p* < 0.001). Hydrocephalus was the most common diagnosis overall, accounting for 27.1% (*n* = 121) of all cases, and was more commonly observed among ward patients compared with ICU patients (30.8% vs. 16.4%; *p* = 0.002). Similarly, normal-pressure hydrocephalus was only observed in the ward group, and the difference between the groups was significant (14.8% vs. 0.0%; *p* < 0.001). Similarly, spastic paraplegia was only identified among the ward patients (4.2% vs. 0.0%; *p* = 0.03). In contrast, intracerebral hematoma was significantly more common in the ICU group (23.3% vs. 1.8%; *p* < 0.001). Subarachnoid hemorrhage was also significantly more common in the ICU group compared to the ward group (12.9% vs. 3.3%; *p* < 0.001). No significant differences were found between the groups regarding intracranial mass, hydrocephalus with meningitis, lumbar and other intervertebral disc disorders, pseudotumor cerebri, subdural hemorrhage, and other diagnoses.

Among the 447 patients included in the study, 103 patients were diagnosed with intracranial masses. Histopathological records were available for 86 of these patients, and the distribution of histopathological diagnoses according to the WHO 2021 Classification of Central Nervous System Tumors is presented in Table 2. Histopathological evaluation revealed a broad spectrum of intracranial lesions, including neoplastic and non-neoplastic entities. Meningiomas represented the most commonly identified tumor category (*n* = 16; 18.6%), followed by adult-type diffuse gliomas (*n* = 15) and embryonal tumors (*n* = 15). Other commonly observed categories included sellar region tumors and metastatic tumors (both *n* = 7; 8.1%). Glioma-related lesions, including adult-type diffuse gliomas, circumscribed astrocytic gliomas, pediatric-type diffuse gliomas, and other astrocytic/glial tumors, constituted an important proportion of the intracranial lesions evaluated. Less commonly observed diagnoses included ependymal tumors, glioneuronal tumors, nerve sheath tumors, mesenchymal non-meningothelial tumors, and non-neoplastic cystic lesions.

Patient-level screening outcomes and microbiological characteristics of isolates according to hospital location (ward vs. ICU) are given in Table 3. Overall, microbiological cultures were positive in 77 patients (17.2%; 95% CI: 14–21%), whereas 370 patients (82.8%; 95% CI: 79–86%) had negative culture results. Culture positivity was significantly higher among ICU patients compared with ward patients (30.2% vs. 12.7%, respectively; *p* < 0.001). Among the 77 culture-positive patients, bacterial growth was detected in 74 (96.1%), whereas yeast was detected in three (3.9%). The distribution of bacterial and yeast isolates did not differ significantly between ICU and ward patients (*p* = 0.59). Three yeast isolates were identified exclusively from initial cultures. One isolate was recovered from a ward patient and was identified as *Candida ciferrii*, whereas two isolates were recovered from ICU patients: one was a *Candida albicans* isolate and the other was reported as *Candida* spp. Among the culture-positive patients, a total of 95 bacterial isolates were obtained, comprising initial isolates (77.9%), follow-up isolates (13.7%), and persistent isolates (8.4%). The isolate dynamic profile showed a significant difference between the ward and intensive care unit (ICU) groups (*p* = 0.008). The initial isolate rate was significantly different: 85.4% in the ward group and 70.2% in the ICU group (*p* = 0.008). The follow-up isolate rate was 14.6% in the ward group and 12.8% in the ICU group, while there was no persistent isolate in the ward group, in comparison to 17% in the ICU group. In contrast, the Gram-stain profile differed significantly between the groups (*p* = 0.002). While Gram-negative isolates were more common in the ICU group compared to the ward group (70.2% vs. 37.5%), Gram-positive isolates were more dominant in the ward group (62.5% vs. 29.8%).

**Figure 1:**
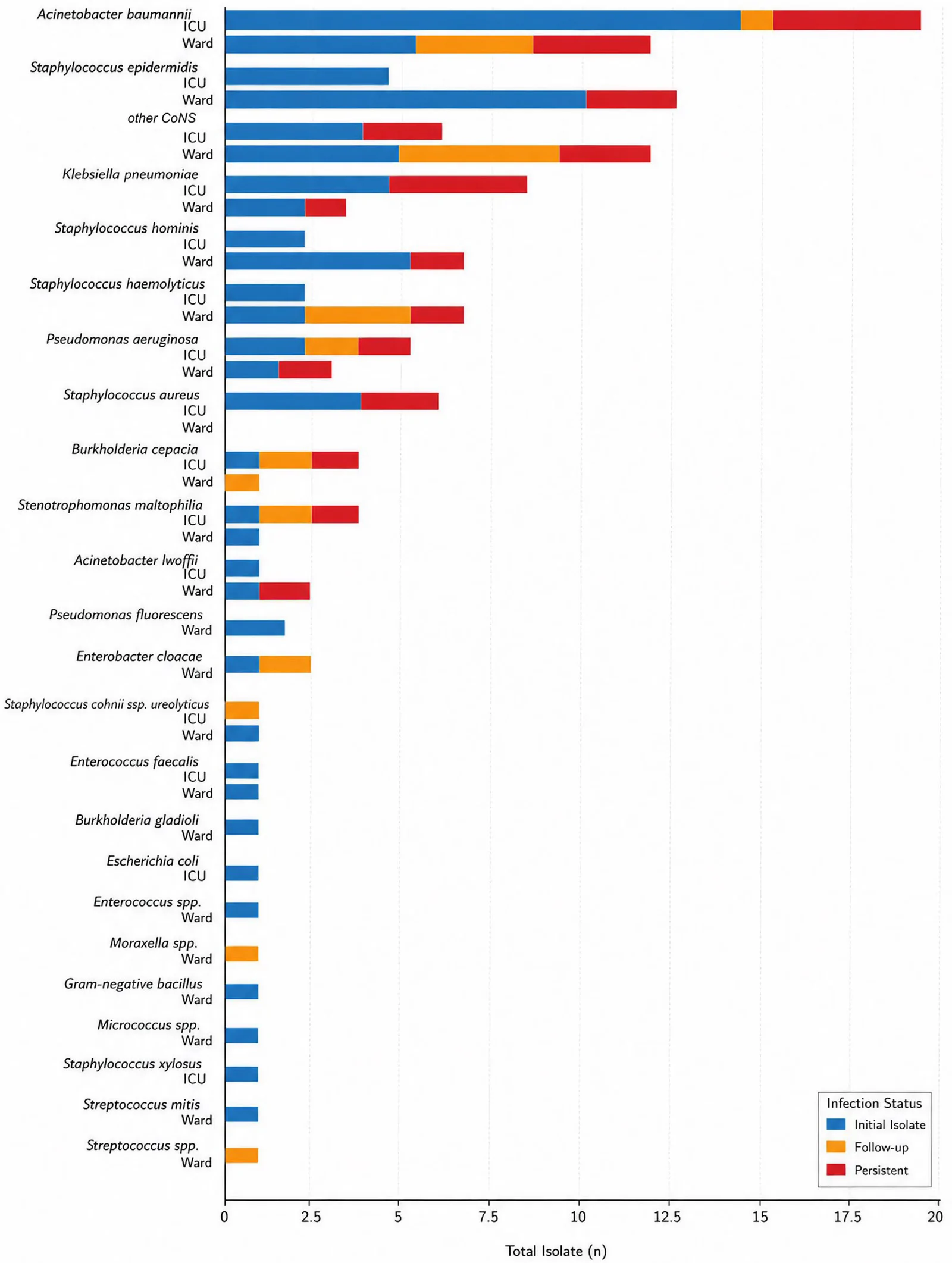
Stratified distribution of CSF bacterial isolates across ward and ICU settings.

A total of 95 bacterial CSF isolates were included in the final comparative analysis after excluding fungal species (*n* = 3) (Figure 1). The following bacterial species were identified: Gram-negative (*A. baumannii: n* = 26; *Acinetobacter lwoffii: n* = 2; *Enterobacter cloacae: n* = 2; *K. pneumoniae: n* = 7; *P. aeruginosa: n* = 4; *Pseudomonas fluorescens: n* = 2; *Pseudomonas* spp.: *n* = 1; *Burkholderia cepacia: n* = 2; *E. coli: n* = 1; Moraxella spp.: *n* = 1; *Stenotrophomonas maltophilia: n* = 3) and Gram-positive (*Staphylococcus epidermidis: n* = 12; *Staphylococcus hominis: n* = 7; *Staphylococcus haemolyticus: n* = 6; *S. aureus: n* = 4; *Streptococcus mitis: n* = 4; *Staphylococcus xylosus: n* = 1; *Staphylococcus cohnii* subsp. *urealyticus*: *n* = 2; *Enterococcus faecalis*: *n* = 2; *Enterococcus* spp.: *n* = 1; *Micrococcus* spp.: *n* = 1; other CoNS: *n* = 6). *A. baumannii* was found predominantly within the ICU setting, which demonstrated the most persistent infection dynamics in the entire cohort, in which 14 were obtained as initial isolates, five as persistent and one as a follow-up isolate. This sustained phenotypic persistence was entirely absent in the ward settings, where *A. baumannii* presentation was rare. Concurrently, *K. pneumoniae* emerged as the second most common Gram-negative pathogenic strain in the ICU. In contrast, Gram-positive isolates were predominantly obtained from patients in the ward setting, with *S. epidermidis*, *S. haemolyticus* and *S. hominis* being the most commonly detected species. While *S. epidermidis* isolates were found to show non-persistent baseline colonization in both settings, *S. haemolyticus* and *S. hominis* exhibited a strict, localized link to the ward setting.

Because AST panels differed between bacterial species, resistance analysis was limited to major antibiotic groups with sufficient test numbers; the results are presented in Table 4. For Gram-negative CSF isolates, carbapenem resistance was observed. Meropenem was tested in 22 isolates, with 40.9% (n = 9) showing resistance, 9.1% (n = 2) showing intermediate susceptibility, and 50% (n = 11) being susceptible. For aminoglycosides, amikacin showed a resistance rate of 45.5% (n = 5/11). Colistin, used as a last-resort antibiotic, remained effective in most cases, with only one isolate found to be resistant. For Gram-positive isolates, resistance to several commonly used antibiotics was common. The clindamycin resistance rate was 56.2% (n = 9/16), and that of tetracycline resistance was high at 83.3% (n = 5/6). Gentamicin showed limited effectiveness, with a 42.9% (n = 3/7) resistance rate. In contrast, glycopeptides remained highly effective, as all tested isolates (n = 6) were susceptible to teicoplanin.

## 4. Discussion

Although ICU patients demonstrated a slightly higher mean age compared with ward patients, this difference did not reach statistical significance in our study. Nevertheless, previous studies have similarly reported that elderly patients are more likely to require ICU-level care due to reduced physiological reserve and increased susceptibility to postoperative complications and healthcare-associated infections [21]. However, the absence of a statistically significant difference in our cohort suggests that age alone may not be the primary determinant of ICU admission in CNS infection cases, and that clinical severity, underlying neurological diagnosis, and acute disease processes may play a more dominant role.

An important strength of the present study is that, although it was conducted at a single tertiary referral center in southern Türkiye, the microbiological profile observed closely aligns with data from other regions across the country. Previous surveillance studies involving neurosurgical and CSF samples from major cities, including İstanbul, Ankara, Diyarbakır, and İzmir, consistently highlight *A. baumannii*, CoNS, *K. pneumoniae*, and *P. aeruginosa* as the primary pathogens causing healthcare-associated CNS infections [17,18,19]. Similarly, our results revealed that *A. baumannii* is most prevalent in intensive care units, whereas CoNS predominates in general wards. This pattern confirms that these pathogens reflect a nationwide epidemiological trend in Turkish neurosurgery rather than center-specific issues. Although exact frequencies varied slightly between hospitals due to local differences in patient demographics, clinical practices, and infection control protocols, the overall bacterial spectrum is remarkably similar. In neurocritical care settings, *A. baumannii* has been recognized as an important cause of postoperative meningitis and device-associated CNS infections, especially following external ventricular drainage or other invasive neurosurgical procedures [15,22]. Similarly, other Gram-negative pathogens identified in our study, including *K. pneumoniae*, *P. aeruginosa*, and *E. cloacae*, were more commonly observed among critically ill patients. These organisms are well-established healthcare-associated pathogens and are commonly linked to prolonged hospitalization, invasive devices, prior antimicrobial exposure, and ICU-related transmission dynamics [23,24]. The occurrence of fungal isolates was low in our cohort, with *Candida* species identified at a low frequency in both ICU and ward populations. In contrast, CNS, *S. epidermidis*, and other commensal skin flora were more prevalent in ward patients. Fungal pathogens, including *Candida* species, were identified in both cohorts at lower frequencies, showing no strong ICU-specific predominance. Consistent with our findings, only a single Candida albicans strain was isolated from CSF samples during three year period in another Turkish hospital [16].

A notable finding in our study was that there was no significant correlation between the patients’ primary neurosurgical diagnosis (e.g., hydrocephalus, intracranial mass, or cerebral hemorrhage) and the specific bacterial species isolated from the CSF. From a clinical and epidemiological perspective, this finding has important implications. It suggests that the development of bacterial infections in neurosurgical patients is not mainly related to the type of primary disease, but rather to common healthcare-associated risk factors. However, this study noted a predominance of Gram-positive organisms in the hydrocephalus cohort, reflecting the pathophysiological mechanism of device-associated neurosurgical infections, as patients with hydrocephalus and intracranial masses are often exposed to similar invasive neurosurgical procedures, including EVD placement, shunt use, and intracranial pressure monitoring [25,26]. These devices may increase the risk of infection by allowing bacteria to enter and form biofilms on their surfaces. It is well known that these biomaterials are highly susceptible to colonization by commensal Gram-positive cocci, especially *S. epidermidis* and *S. aureus*, which possess specialized microbial surface components that help them adhere to prosthetic surfaces and thus facilitate biofilm formation [27,28]. In contrast, Gram-negative bacteria were more common among patients with intracranial masses in the current study. Brain tumor surgery is frequently associated with prolonged operative times, intensive care unit stays, repeated manipulation, and the use of invasive neurosurgical devices, all of which are recognized risk factors for healthcare-associated infections caused by opportunistic nosocomial pathogens, including *A. baumannii* and *K. pneumoniae* [29,30].

Due to the utilization of different phenotypic AST panels for different bacterial species, resistance tracking was kept to restricted core agent classes in the current study. The identification of a high meropenem resistance rate among Gram-negative pathogens highlights a concerning therapeutic challenge. In neurocritical care, carbapenems are commonly used as the main empirical treatment for suspected post-craniotomy meningitis or ventriculitis because they have a broad antibacterial spectrum and can reach effective levels in the meninges during inflammation [26,31,32,33]. The high rate of amikacin resistance further limits the available treatment options and reduces the effectiveness of commonly used beta-lactam/aminoglycoside combinations. Consistent with our finding, a large Turkish study that included 15,519 CSF samples reported resistance rates of 40% to amikacin and 58% to meropenem among *A. baumannii* strains [34]. Our findings indicate that high-risk neuro-ICU patients with severe meningitis may require broader empirical antimicrobial coverage, including last-resort agents such as colistin or newer cephalosporin–beta-lactamase inhibitor combinations, until microbiological results are available. These results are in agreement with recent studies from Türkiye, which have also reported high resistance levels against last-resort antimicrobial agents, including carbapenems and colistin, among Gram-negative isolates [9].

High resistance rates to clindamycin and tetracycline make these drugs unsuitable for secondary treatment. In contrast, glycopeptides showed effectiveness against all isolates. This supports international guidelines, which indicate that despite the global increase in multidrug-resistant Gram-positive bacteria, glycopeptides such as vancomycin and teicoplanin remain reliable options for empirical treatment of suspected device-related or shunt infections in surgical patients [26]. Supporting this, approximately 90% of CoNS isolates were methicillin-resistant, whereas vancomycin retained full activity against all tested staphylococcal and enterococcal isolates [9].

## 5. Conclusions

This study demonstrates that healthcare-associated and device-related factors, rather than the primary neurosurgical diagnosis, play an important role in the development of central nervous system infections in neurosurgical patients. The microbiological profile was found to be dominated by *A. baumannii*, CoNS, and *K. pneumoniae*, with a clear shift toward Gram-negative pathogens in critically ill ICU patients. In addition, significant antimicrobial resistance was observed, particularly among Gram-negative isolates, limiting the effectiveness of commonly used empirical therapies and supporting the continued role of carbapenems and glycopeptides, while also highlighting the potential need for last-resort agents in selected high-risk cases.

However, this study has important limitations. Due to the retrospective design, clinical outcome data such as patient survival, long-term neurological status, and functional recovery could not be systematically evaluated. This limitation was mainly influenced by the major earthquake that occurred in Hatay in 2023, which disrupted hospital infrastructure and made complete follow-up of patients impossible. As a result, outcome-based correlations could not be fully assessed. Furthermore, because this was a single-center study, the findings may only be applicable to tertiary care centers with comparable neurosurgical patient populations, referral patterns, antimicrobial prescribing practices, and infection-control policies. Despite these limitations, our findings provide valuable epidemiological and microbiological insights into neurosurgical CNS infections. They highlight the importance of local resistance surveillance and suggest that future well-designed, prospective, and multicenter studies are necessary to better define risk factors, optimize empirical treatment strategies, and improve infection control in neurocritical care settings.

## Figures and Tables

**Table 1:** Clinical and demographic characteristics of patients according to admission setting.

Characteristic	Total (*n* = 447)	Ward (*n* = 331)	ICU (*n* = 116)	*p*-Value
Age at admission (years), mean ± SD	36.1 ± 14.8	35.5 ± 14.6	37.7 ± 15.2	0.41 *
**Gender**				
Female	195 (43.6%)	150 (45.3%)	45 (38.8%)	0.23
Male	252 (56.4%)	181 (54.7%)	71 (61.2%)	
**Diagnosis**				**<0.001 †**
Hydrocephalus	121 (27.1%)	102 (30.8%)	19 (16.4%)	**0.002 ‡**
Hydrocephalus with meningitis	12 (2.7%)	8 (2.4%)	4 (3.4%)	0.52 ‡
Intracerebral hematoma	33 (7.4%)	6 (1.8%)	27 (23.3%)	**<0.001 ‡**
Intracranial mass	103 (23.0%)	73 (22.1%)	30 (25.9%)	0.44 ‡
Lumbar and other intervertebral disc disorders	9 (2.0%)	9 (2.7%)	0 (0.0%)	0.12 ‡
Normal-pressure hydrocephalus	49 (11.0%)	49 (14.8%)	0 (0.0%)	**<0.001 ‡**
Others	55 (12.3%)	40 (12.1%)	15 (12.9%)	0.87 ‡
Pseudotumor cerebri	11 (2.5%)	11 (3.3%)	0 (0.0%)	0.07 ‡
Spastic paraplegia	14 (3.1%)	14 (4.2%)	0 (0.0%)	**0.03 ‡**
Subarachnoid hemorrhage	26 (5.8%)	11 (3.3%)	15 (12.9%)	**<0.001 ‡**
Subdural hemorrhage	14 (3.1%)	8 (2.4%)	6 (5.2%)	0.21 ‡

* Student’s *t* test; † chi-square/exact test; ‡ z test; *p* < 0.05 was considered statistically significant.

**Table 2:** Histopathological classification of intracranial lesions according to the WHO CNS Classification.

WHO 2021 Category	*n* (%)	Histopathological Diagnoses Included
Embryonal Tumors	15 (17.4%)	Medulloblastoma, classic medulloblastoma, nodular/desmoplastic medulloblastoma, primitive neuroectodermal tumor (PNET)
Adult-Type Diffuse Gliomas	15 (17.4%)	Diffuse astrocytoma, oligodendroglioma, anaplastic oligodendroglioma, glioblastoma/glioblastoma multiforme
Circumscribed Astrocytic Gliomas	2 (2.3%)	Pilocytic astrocytoma
Pediatric-Type Diffuse Gliomas	5 (5.8%)	Brainstem glioma, high-grade glial tumor
Other Astrocytic/Glial Tumors	6 (7.0%)	Astrocytoma, glial tumor, reactive gliosis/low-grade glial tumor, anaplastic astrocytoma
Ependymal Tumors	1 (1.2%)	Ependymoma
Glioneuronal and Neuroepithelial Tumors	1 (1.2%)	Neuroepithelial tumor
Meningiomas	16 (18.6%)	Meningothelial, transitional, fibroblastic, metaplastic, typical, and atypical meningioma
Cranial and Peripheral Nerve Sheath Tumors	2 (2.3%)	Schwannoma, ancient schwannoma
Sellar Region Tumors	7 (8.1%)	Pituitary adenoma, GH-secreting pituitary adenoma, pituitary macroadenoma, prolactinoma, craniopharyngioma, adamantinomatous craniopharyngioma
Mesenchymal Non-Meningothelial Tumors	1 (1.2%)	Chondroid chordoma
Metastatic Tumors	7 (8.1%)	Metastatic adenocarcinoma, malignant epithelial tumor metastasis, germ cell tumor metastasis
Germ Cell Tumors	1 (1.2%)	Teratoma with somatic malignant transformation
Lymphomas and Hematopoietic Tumors	1 (1.2%)	High-grade B-cell non-Hodgkin lymphoma
Other Malignant Tumors	3 (3.5%)	Adenoid cystic carcinoma, high-grade round-cell malignant tumor
Non-Neoplastic and Cystic Lesions	3 (3.5%)	Epidermoid cyst, epidermal cyst, no evidence of neoplasia

Note: Percentages were calculated among patients with evaluable histopathological results. Among 103 patients classified as having an intracranial mass, 86 had evaluable histopathological results.

**Table 3:** Distribution of culture positivity, isolate characteristics, and Gram-stain profiles among neurosurgical patients admitted to a ward and the ICU.

Characteristic	Total Cohort (*n* = 447) *n* (%) [95% CI]	Ward (*n* = 331) *n* (%) [95% CI]	ICU (*n* = 116) *n* (%) [95% CI]	*p*-Value
Patient-level culture status				**<0.001**
Negative	370 (82.8%) [79%, 86%]	289 (87.3%) [83.3%, 90.5%]	81 (69.8%) [60.9%, 77.4%]	
Positive	77 (17.2%) [14%, 21%]	42 (12.7%) [9.5%, 16.7%]	35 (30.2%) [22.6%, 39.1%]	
Microorganism in initial culture-positive patients				0.59
Bacteria	74 (96.1%) [89.2%, 98.7%]	41 (97.6%) [87.7%, 99.6%]	33 (94.3%) [81.4%, 98.4%]	
Yeast	3 (3.9%) [1.3%, 10.8%]	1 (2.4%) [0.4%, 12.3%]	2 (5.7%) [1.6%, 18.6%]	
Isolate dynamics profile				**0.008**
Initial isolate	74 (77.9%) [68.6%, 85.1%]	41 (85.4%) [72.8%, 92.8%]	33 (70.2%) [56.0%, 81.3%]	
Follow-up isolate	13 (13.7%) [8.2%, 22.0%]	7 (14.6%) [7.2%, 27.2%]	6 (12.8%) [6.0%, 25.2%]	
Persistent isolate	8 (8.4%) [4.3%, 15.7%]	0 (0.0%) [0.0%, 7.4%]	8 (17.0%) [8.9%, 30.1%]	
Gram-stain phenotypic profile				**0.001**
Gram-negative	51 (53.7%) [43.7%, 63.4%]	18 (37.5%) [25.2%, 51.6%]	33 (70.2%) [56.0%, 81.3%]	
Gram-positive	44 (46.3%) [36.6%, 56.3%]	30 (62.5%) [48.4%, 74.8%]	14 (29.8%) [18.7%, 44.0%]	

CI = confidence interval. Percentages and 95% CIs were calculated using the Wilson score method based on the respective denominator of each clinical section (total cohort, *n* = 447; culture-positive patients, *n* = 77; total bacterial strains, *n* = 95). Chi-square/exact test; *p* < 0.05 was considered statistically significant.

**Table 4:** Antimicrobial resistance patterns of principal CSF isolates.

Organism Type/Antibiotic	Susceptible, *n* (%)	Intermediate, *n* (%)	Resistant, *n* (%)	Total Tested (*n*)
**Gram-Negative Pathogens**				
Meropenem	11 (50.0%)	2 (9.1%)	9 (40.9%)	22
Amikacin	5 (45.5%)	1 (9.1%)	5 (45.5%)	11
Colistin	5 (83.3%)	0 (0.0%)	1 (16.7%)	6
**Gram-Positive Pathogens**				
Clindamycin	7 (43.8%)	0 (0.0%)	9 (56.2%)	16
Tetracycline	1 (16.7%)	0 (0.0%)	5 (83.3%)	6
Gentamicin	4 (57.1%)	0 (0.0%)	3 (42.9%)	7
Teicoplanin	6 (100.0%)	0 (0.0%)	0 (0.0%)	6

AST results were deduplicated at the patient–organism–antibiotic level. Only antibiotics with a cumulative test count of *n* ≥ 4 within the tested isolates are presented to maintain statistical representativeness. Percentages were calculated using the number of tested isolates for each antimicrobial agent as the denominator.

## Data Availability

Dataset available on request from the authors.

## Abbreviations

The following abbreviations are used in this manuscript:

CNS	Central nervous system
ICU	Intensive care unit
CSF	Cerebrospinal fluid
MDR	Multidrug-resistant
CoNS	Coagulase-negative staphylococci
AMR	Antimicrobial resistance
AST	Antimicrobial susceptibility testing
